# Use of Non-Destructive Measurements to Identify Cucurbit Species (*Cucurbita maxima* and *Cucurbita moschata*) Tolerant to Waterlogged Conditions

**DOI:** 10.3390/plants9091226

**Published:** 2020-09-18

**Authors:** Hsin-Hung Lin, Kuan-Hung Lin, Meng-Yuan Huang, Yi-Ru Su

**Affiliations:** 1 Department of Horticulture and Biotechnology, Chinese Culture University, Taipei 11114, Taiwan; rlin@faculty.pccu.edu.tw (K.-H.L.); a6221389@g.pccu.edu.tw (Y.-R.S.); 2 Department of Life Sciences, National Chung-Hsing University, Taichung 40227, Taiwan; hmy6@nchu.edu.tw

**Keywords:** chlorophyll fluorescence, cucurbit, spectral reflectance, squash, waterlogging tolerance

## Abstract

Limited information is available regarding the physiology of squash plants grown under waterlogging stress. The objectives of this study were to investigate the growth and physiological performances of three cucurbit species, *Cucurbita maxima* cultivar (cv.) OK-101 (OK) and *Cucurbita moschata* cv. Early Price (EP) and Strong Man (SM), in response to waterlogging conditions, and to develop a precise, integrated, and quantitative non-destructive measurement of squash genotypes under stress. All tested plants were grown in a growth chamber under optimal irrigation and growth conditions for a month, and the pot plants were then subjected to non-waterlogging (control) and waterlogging treatments for periods of 1, 3, 7, and 13 days (d), followed by a 3-d post-waterlogging recovery period after water drainage. Plants with phenotypes, such as fresh weight (FW), dry weight (DW), and dry matter (DM) of shoots and roots, and various physiological systems, including relative water content (RWC), soil and plant analysis development (SPAD) chlorophyll meter, ratio of variable/maximal fluorescence (*Fv/Fm*), quantum photosynthetic yield (YII), normalized difference vegetation index (NDVI), and photochemical reflectance index (PRI) values, responded differently to waterlogging stress in accordance with the duration of the stress period and subsequent recovery period. When plants were treated with stress for 13 d, all plants exhibited harmful effects to their leaves compared with the control, but EP squash grew better than SM and OK squashes and exhibited stronger tolerance to waterlogging and showed less injury. Changes in the fresh weight, dry weight, and dry matter of shoots and roots indicated that OK plants suffered more severely than EP plants at the 3-d drainage period. The values of RWC, SPAD, *Fv/Fm*, YII, NDVI, and PRI in both SM and OK plants remarkably decreased at waterlogging at the 13-d time point compared with controls under identical time periods. However, the increased levels of SPAD, *Fv/Fm*, YII, NDVI, and PRI observed on 7 d or 13 d of waterlogging afforded the EP plant leaf with improved waterlogged tolerance. Significant and positive correlations were observed among NDVI and PRI with SPAD, *Fv/Fm*, and YII, indicating that these photosynthetic indices can be useful for developing non-destructive estimations of chlorophyll content in squashes when screening for waterlogging-tolerant plants, for establishing development practices for their cultivation in fields, and for enhanced cultivation during waterlogging in frequently flooded areas.

## 1. Introduction

The Cucurbitaceae (cucurbit) family contains 825 species derived from tropical and subtropical regions. Among them, 26 species are cultivated as vegetables [1]. The squash plant, native to Central and South America, is a creeping, climbing, herbaceous, monoecious, and annual plant [2]. The squash cultivar, *Cucurbita maxima* (Cma), is commonly referred to as winter squash, and is being used successfully as a cold-hardy rootstock that is resistant to chilling stress for winter production of bitter melon (*Momordica charantia*) [3]. In addition, *Cucurbita moschata* (Cmo) squash cultivar is adapted to warmer climates, mostly grown in tropical and subtropical regions, and generally more tolerant of hot and humid weather than Cma [4]. However, waterlogged soil with poor drainage causes most quash cultivars to rot and to display yellow and cupping leaves [5].

Global climate change is strongly associated with variations in heavy rainstorms and waterlogging events, which can leave soil saturated for days [6]. Air pockets in soil become filled with water during saturation, thus creating low-oxygen conditions followed by anoxia. When roots are submerged, anoxic conditions inhibit aerobic respiration, yielding low energy, thereby causing roots to transport smaller amounts of water to the shoots [7]. Waterlogging conditions result in many physiological changes, such as leaf necrosis, chlorosis, and defoliation, as well as changes in various plant growth regulators and signaling molecules of flood-sensitive crops [8]. Physiological processes, including an increase in chlorophyll (Chl) breakdown and lower membrane permeability, peroxidation, petiole epinasty, photosynthetic performance, and stomatal conductance, are related to waterlogging and can influence differences in tolerance to waterlogging and these processes can greatly influence plant survival [9,10]. Moreover, waterlogging stress also reduces the ability of photosynthesis to utilize incident photons, leading to photoinhibition, a concomitant reduction in the quantum yield of photochemistry, and a decrease in chlorophyll fluorescence (ChlF) [11]. Studying cucurbit species’ differential growth patterns in response to waterlogging will provide the physiological bases for improving their tolerance to stresses, but visual observations frequently result in experimental errors, whereas destructive measurements damage plants and make further experiments nearly impossible. Thus, the use of non-destructive automated phenotyping may enable more accurate quantification of symptoms, enhancing the possibility of making progress in genetic gain. The ChlF measurement, such as the maximal quantum yield of photosystem II (PSII) photochemistry, is a non-invasive technique and has been widely used in a range of photosynthetic tissues for studying functional changes in the photosynthetic apparatus under environmental stress [12]. Reflectance spectroscopy is another underexploited, non-invasive technique that can be used in physiological studies; it is simple, rapid, and non-destructive in nature [13]. Stress can lead to changes in reflectance spectra, and these changes can be used to calculate different vegetation indices, such as normalized difference vegetation index (NDVI) and photochemical reflectance index (PRI). These changes in reflectance spectra have also been linked to photosynthetic light-use efficiency [14,15]. Furthermore, NDVI was applied for estimating the water content (WC) associated with the water absorption band centered at 1240 nm [16]. Many theoretical models based on leaf reflectance were developed to predict leaf Chl content, WC, and other variables associated with the vegetative structure [17]. Hence, these spectral reflectance indices might be useful for measuring leaf pigments and plant growth under waterlogging treatments when developing indices for non-destructive estimations.

Waterlogging induced by large rainfall events is a major risk to fresh-market squash production in Taiwan, and most squash cultivars are not capable of tolerating waterlogging stress when the soil is saturated with water due to typhoon-caused heavy rains during the summer. To overcome this problem, some squash growers use seedlings grafted onto selected sponge gourd (*Luffa cylindrica*) rootstocks to reduce the loss of squashes in wet summer seasons in Taiwan. Nevertheless, studies of the responses of the physiological systems of squash to waterlogging stress are scarce. Up to date, the physiological mechanisms of leaf ChlF and spectral reflectance indices of acquired tolerance to waterlogging in Cma and Cmo plants have not been found in any literature. The authors hypothesized that some of the ChlF components and spectral reflectance parameters might exhibit distinguishable differences between Cma and Cmo plants under different waterlogging treatments, and these indices would be changed in different genotypes with different Chl contents in response to different waterlogging conditions. Furthermore, the development of waterlogging-tolerant cultivars is a good strategy for improving the production of squash in summer, but breeding of squash with broad waterlogging stress resistance is hampered due to the lack of reliable, easy-to-measure, and practical selection tools, such as physiological markers. The use of such markers is currently problematic because our knowledge of the physiology behind successful selection of a waterlogging-tolerant squash is still very limited. The long-term goal of our work is to help breed a waterlogging-tolerant squash to be grown in wetlands or other areas subject to short and intense rainfall events. The objectives of this work were to study the non-destructive ChlF and spectral reflectance properties to identify waterlogging tolerance of squashes under waterlogged conditions, and these indices can be considered as selection indexes for examining the growth of squash plants at specific and optimal waterlogging treatments. Consequently, identifying the non-destructive parameters that control waterlogging-induced tolerance in squash plants is a major prerequisite for improving crop yields. The responses of the photosynthetic apparatus to waterlogging are probably critical for activating and integrating stress defense mechanisms, and thus may play important roles in the process of reducing waterlogging stress.

## 2. Results

### 2.1. Waterlogging Effects on Growth Traits in Squashes

Table 1 illustrates how the dry weight (DW) and fresh weight (FW) of shoots and roots differed in the three genotypes of squash after 13 d of waterlogging treatments followed by 3-d recovery after drainage, and specific genotype responses to stress were correlated with resistance to waterlogging stress. The shoot FW of OK-101 (OK) cultivar with the 3-d recovery treatment was significantly lower (7.16 ± 1.16 g) than that of Early Price (EP) cultivar in all treatments (11.01 ± 1.17 g to 8.98 ± 1.29 g). When all waterlogging treatments across genotypes were compared, significantly higher root FW of both EP and Strong Man (SM) plants with 3-d recovery was observed (3.16 ± 0.74 g to 3.73 ± 0.35 g) compared with OK plants under 3-d recovery (1.62 ± 0.29 g) and control under identical time periods (1.12 ± 0.34 g), suggesting that both EP and SM plants were more tolerant to waterlogging than OK plants. Similar trends and rates of DW in shoots and roots were also observed in both EP and SM plants, and maximal increases occurred during 3-d recovery (1.79 ± 0.20 g to 1.60 ± 0.51 g (shoots) and 0.27 ± 0.05 g to 0.28 ± 0.11 g (roots)) compared with other treatments. Significant increases in the dry matter (DM) of shoots of all cultivars under waterlogging treatments for 13 d (13.45 ± 0.57% to 16.87 ± 3.14%) and for 3-d recovery (14.54 ± 2.34% to 22.84 ± 2.86%) were observed compared with their control under identical time periods (7.73 ± 0.72% to 9.56 ± 1.24% and 10.06 ± 1.00% to 18.45 ± 2.91%, respectively), implying that the surviving plants subjected to waterlogging would accumulate shoot biomass. Waterlogging stress over time caused a change in root DM of different genotypes. The DM of EP squash roots was found to be higher after recovery (11.58 ± 1.69%) and in its simultaneously paired control (8.53 ± 1.62%) compared with OK squash after recovery (6.55 ± 1.10%) and its simultaneously-paired control (5.92 ± 1.54%), indicating that when the stress was removed, EP plants recovered faster and accumulated more root biomass than OK plants. In addition, the genotypic response to root/shoot ratio under waterlogging treatments was consistent, and followed a response pattern similar to that of DW. EP and SM plants exhibited a significantly higher root/ shoot FW ratio (42.16 ± 4.44% to 42.97 ± 4.32%) and root/shoot DW ratio (19.33 ± 3.38% to 15.77 ± 3.21%) than OK plants (20.29 ± 2.16% of root/shoot FW and 8.02 ± 1.47% of root/shoot DW) following the 3-d recovery period, indicating that root FW and DW increased in both EP and SM plants subjected to waterlogging. 

**Figure 1:**
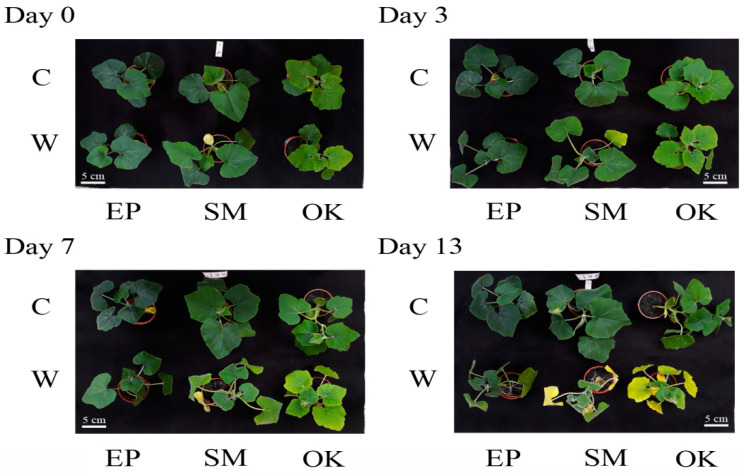
Appearance of EP, SM, and OK squash plants grown under optimally irrigated (control, C) or waterlogging (W) conditions for 0 (non-waterlogging), 3, 7, and 13 d periods. Bar indicates 5 cm.

Figure 1 shows that all leaves visually appeared to be green and healthy at the beginning and at 3 d of waterlogging. However, for waterlog treatments longer than 3 d, all genotypes exhibited different patterns in which SM and OK plants were progressively curled and folded, appeared necrotic, or wilted over the course of time, and the lower leaves of both cultivars visually showed epinasty and senescence at 7 d of waterlogging, but most leaves looked green and healthy in the control. Particularly, the entire OK plants looked retarded, rolled, impaired, and of small size relative to all of the other plants at 13 d of waterlogging treatment. Consequently, waterlogging stress exhibited a harmful effect on OK plant leaves. After 13 d of waterlogging, most SM and OK plants were severely damaged, preventing the ability to collect leaf samples for the analysis of physiological parameters. In many cases, both SM and OK plants that survived waterlogging died after the stress was removed. As waterlogging became more severe, the differences between the three cultivars became more distinct. Notably and of great interest, EP plants grew better than SM and OK plants after 13 d of waterlogging treatment, exhibiting stronger tolerance to waterlogging and showing less injury.

### 2.2. Physiological Responses of Squash Plants to Waterlogging Stress

**Figure 2:**
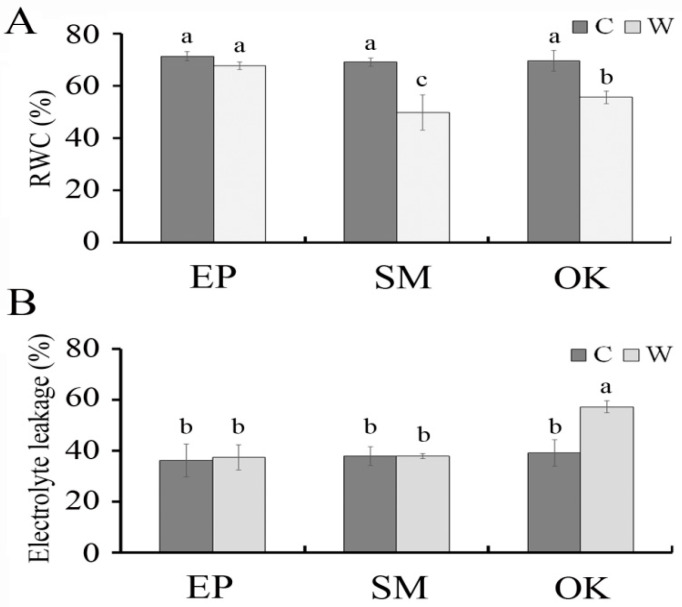
Effects of waterlogging on (**A**) relative water content (RWC, %) and (**B**) electrolyte leakage (EL, %) in EP, SM, and OK plants. Plants were grown under optimally irrigated (control, C, black bars) or waterlogging (W, white bars) conditions for 13 days. Means followed by different letters are significantly different at *p* ≤ 0.05 by least significant difference (LSD). Vertical bars indicate the standard error (*n* = 4).

Figure 2 shows the varied responses of all cultivars toward different relative water content (RWC) and electrolyte leakage (EL) values of leaves for the 13-d waterlogging treatment. No significant differences in RWC (67.55–71.27%) and EL (36.01–37.35 %) values were observed between waterlogging and control tests in EP plants. However, in both SM and OK plants, RWC in waterlogging treatments (49.75–55.53%) was significantly lower than that in controls (69.03–69.48%), suggesting that SM and OK plants were less tolerant to waterlogging than EP plants. Moreover, the leaf EL level of the OK cultivar at 13 d of waterlogging (57.16%) was significantly higher than that of the control (39.17%) and other cultivars (37.35–37.84%), indicating that ionic leakage in OK plants increased after waterlogging for 13 d and the plants exhibited intolerance to waterlogging.

Table 2 illustrates that the soil and plant analysis development (SPAD) chlorophyll meter levels of all plants showed no significant differences among control and waterlogging treatments at 0, 1, and 3 d, indicating that SPAD values of genotypes were not affected by 3 d of waterlogging. However, SPAD values of all plants were significantly lower than that of control plants under waterlogging treatment at both 7 d and 13 d of treatment. Different cultivars showed different SPAD responses to waterlogging treatments; the EP plant showed significantly higher SPAD than SM and OK plants under waterlogging treatment at both 7 d and 13 d of treatment, suggesting that the EP was more tolerant to waterlogging than SM and OK. Moreover, the SPAD levels of EP plants were accumulated at different rates from 0 d (46.24 ± 3.27 and 46.54 ± 3.36) to 13 d (51.78 ± 4.27 and 41.04 ± 3.22) of control and waterlogging, respectively. However, SPAD values in both SM and OK plants progressively decreased from 0 to 7 d (42.02 ± 3.57 to 30.59 ± 3.05 in the SM plant and 29.41 ± 3.90 to 23.75 ± 2.55 in the OK plant) of waterlogging, and then rapidly dropped after 13 d (10.35 ± 2.02 in the SM plant and 6.45 ± 1.16 in the OK plant) of waterlogging treatment.

Table 3 demonstrates comparisons of ratio of variable/maximal fluorescence (*Fv/Fm*) and quantum photosynthetic yield (YII) values under control and waterlogging treatment at each time point in leaves of all cultivars. When genotypes across waterlogging treatments were compared, *Fv/Fm* values in all tested genotypes remained similar under both control and waterlogging treatments at 0 (non-waterlogging), 1, 3, and 7 d, and ranged from 0.768 ± 0.033 to 0.810 ± 0.007, suggesting that the *Fv/Fm* values of genotypes were not affected by 7 d of waterlogging. Nevertheless, *Fv/Fm* values in SM and OK squashes under waterlogging treatment at 13 d were significantly lower than that in other treatments of the tested plants, and the lowest value (0.347 ± 0.003) was found in OK squash after 13 d of waterlogging. YII values had similar patterns to Fv/Fm, and all genotypes remained similar under both control and waterlogging treatments at 0, 1, 3, and 7 d of treatment, except that OK plants under waterlogging treatment at both 3-d and 7-d time points exhibited significantly lower YII levels (0.668 ± 0.030 and 0.644 ± 0.044, respectively) than control under identical time periods (0.724 ± 0.020 and 0.704 ± 0.028, respectively). 

As shown in Table 4, the three squash cultivars possessed different NDVI and PRI values in response to various waterlogging treatments. When all cultivars were compared across all treatments, both EP and SM plants displayed significantly higher NDVI values than OK plants in control and waterlogging treatments at 0, 1, and 3 d of treatment. Nevertheless, SM and OK plants showed significantly lower NDVI levels in the waterlogging treatments at 7 d (0.686 ± 0.021 and 0.376 ± 0.017, respectively) and 13 d (0.648 ± 0.021 and 0.459 ± 0.080, respectively) of treatment than those in the control under identical time periods (0.733 ± 0.017 and 0.727 ± 0.016 for 7 d, and 0.709 ± 0.018 and 0.699 ± 0.020 for 13 d, respectively). The PRI value of green SM plants with waterlogging treatment at 13 d of treatment (0.024 ± 0.013) was significantly lower than that of its control (0.061 ± 0.008) when genotypes were compared across various waterlogging treatment. Significantly lower PRI values were also found in OK plants undergoing 7 d and 13 d of waterlogging (0.064 ± 0.004 and 0.018 ± 0.013, respectively) compared with their simultaneously paired control (0.072 ± 0.004 and 0.063 ± 0.009, respectively).

### 2.3. Relationships Among SPAD, ChlF, and Spectral Reflectance Variables in Squashes

**Figure 3:**
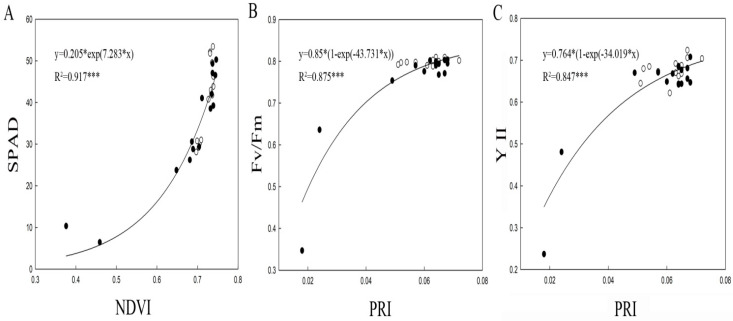
The regression correlations between (**A**) SPAD and NDVI, (**B**) *Fv/Fm* and PRI, and (**C**) YII and PRI in plants under control and waterlogging treatments. Each symbol represents the average of ten plants which were randomly selected from each treatment. Each NDVI or PRI index was calculated using leaf SPAD, *Fv/Fm*, and YII data (*n* = 30) from the model validation datasets. The R^2^ coefficients of NDVI and PRI with leaf SPAD, *Fv/Fm,* and YII were calculated. The white circle represents data from control treatments, whereas the black circle represents data from waterlogging treatments. ***p < 0.001.

To investigate whether spectral reflectance indices were sensitive to SPAD and ChlF components, the coefficients of R^2^ among them were examined by regression. Figure 3 shows the changes in NDVI and PRI of all cultivars under waterlogging conditions over the 13-d period. The leaf SPAD progressively increased with increasing NDVI value, and most plants showed high *Fv/Fm* (0.739–0.376) with high SPAD (53.42–6.45). The regression analysis showed that SPAD was significantly and strongly correlated with leaf NDVI at R^2^ = 0.917 (*p* < 0.001), demonstrating the applicability of NDVI for measuring total Chl contents in response to waterlogging. Moreover, the trend of PRI vs. *Fv/Fm* and YII was similar, and as PRI increased from 0.018 to 0.072, *Fv/Fm* and YII values increased from 0.347 to 0.810 and from 0.237 to 0.724, respectively, in response to waterlogging. Significant correlations of PRI with *Fv/Fm* (R^2^ = 0.875, *p* < 0.001) and YII (R^2^ = 0.847, *p* < 0.001) were also observed in all tested plants under waterlogging conditions, indicating that PRI affected *Fv/Fm* and YII positively.

## 3. Discussion

The determination of the function of an observed response is one of the most complicated issues in plant stress physiology, and plant responses to waterlogging stress are complex phenotypic and physiological phenomena that are highly influenced by environmental factors. Waterlogging leads to a decrease in the efficiency of photosystem II in a plant, followed by a decrease in the growth rate [18]. To survive waterlogging stress, plants have evolved a number of physiological responses. In this study, after 13-d of waterlogging stress, the OK plants appeared epinastic with wilting and loss of photosynthetic capability, and these characteristics did not disappear after plants were removed from stress conditions with 3-d recovery. In general, early flooding (3-d stress) had no harmful effects on plants; yet, with prolonged waterlogging stress, significant waterlogging injury was observed. The waterlogging stress conditions used in this study (0–13 d) influenced plant growth, but these effects were not lethal. The 3-d post-waterlogging (recovery) period could be as injurious as waterlogging itself, in part because senescence-associated processes were initiated in response to the original stress. Plants which are tolerant to waterlogging need to survive or grow during the stress, but they also need to recover after the stress is removed [7]. Thus, observed responses of squashes probably included adaptive responses to waterlogging. We utilized a non-destructive physiological approach to discover the changes in plant growth and photosynthetic profiles of squashes in response to waterlogging stress, and attempted to determine whether these indices could be used on the plants as sensitive metrics for estimating leaf photosynthetic apparatus corresponding to plant growth.

When roots are submerged, the anoxic condition inhibits aerobic respiration, which yields less energy. As the roots translocate less water and nutrients to the leaves, the solutes entering the leaves through the transpiration stream may also decrease. As is known, stomatal closure causes a decrease in internal CO_2_ concentrations, and subsequently, a concomitant decline in photosynthesis can result from the diminished availability of CO_2_ for carbon fixation, leading to senescence and even death of plants [19]. In our study, waterlogging treatments may not have induced stomatal closure and did not affect RWC and EL, but there was a minor decrease in SAPD. Thus, the capability of EP plants to tolerate waterlogging was confirmed by RWC, SPAD, and EL values, which were recorded from the tested plants exposed to waterlogging for 13 d, in comparison to control plants without waterlogging for an identical time period, and those physiological processes may have been associated with this tolerance. Waterlogging stress had a harmful effect on the leaves of the squash variants, and changes in SPAD values might be related to the degree of chlorosis and reduced RWC of the plant leaves during waterlogging. EP plants may have generated the driving force for leaf water charging of cellular water storage capacities, improving the water status of leaves. The mechanism is not clearly understood, but this process might result in more efficient water uptake by plants or a decrease in water loss from plants, or perhaps both, during waterlogging stress. Alternatively, the degree of waterlogging-induced injury seemed to have been the result of the enhancement of EL, and a decline RWC and SPAD values, in SM and OK plants under waterlogging stress. The tested plants showed a progressive decline in growth as the duration of stress increased, indicating that long-term waterlogging stress induced a decline in the growth-related traits of these plants. In particular, the leaves of waterlogged OK plants were epinastic, short, and stunted after 3-d of flooding (Figure 1). These visual symptoms could be used to infer reduced chlorophyll content (CC) when monitoring waterlogging damage. Therefore, increases in SPAD values were observed before leaves became epinastic and senescent under waterlogging stress, and this identified system can be used for rapid monitoring and early detection of waterlogging injury.

The SPAD-502 m is widely used for the rapid, accurate, and non-destructive measurement of CC in leaves. The positive linear relationship between SPAD value and CC was shown by Ling et al. [20]. Along with the visible symptoms, the reduced CC could be used to monitor the damage induced by waterlogging in both EP and OK leaves, and SPAD can help in the advanced interpretations of the photochemical process in plants. Consequently, the SPAD parameter was chosen as an indirect proxy of plant physiology under waterlogging treatment to test the correlation with NDVI in the squashes, and it may be used as a criterion for differentiating the levels of tolerance plants have to waterlogging. SPAD can also provide a quantitative assessment for the easy screening of waterlogging-tolerant EP plants based on their ability to overcome the stress of waterlogging. Chlorophyll function does not decline in stressed waterlogging-tolerant plants, and the positive correlation between the ChlF and SPAD values was reported by Netto et al. [21]. In addition, while studying winter wheat under varying irrigation treatments, Liu et al. [22] reported that both ChlF and NDVI were positively and significantly correlated with the root zone soil moisture for measuring vegetation variations even when the CC was at a high level. Jiménez et al. [23] reported that NDVI can serve as a non-destructive screening approach for the identification of *Brachiaria* hybrids tolerant to waterlogging stress under field conditions. Therefore, the NDVI is more comprehensively applicable for the non-destructive estimation of CC in plant leaves and can indicate the photosynthetic capacity. The long-term goal of our work is to help breed a competitively higher waterlogging-tolerant squash to be grown in wetlands, lowlands, or areas that are prone to flooding, such as where there is natural gleying of the soil or in areas subject to short and intense rainfall. The identification of unique, stress-responsive, non-destructive measurements, such as NVDI and SPAD values, will allow further dissection of the genetic basis of this transgressive performance in the offspring of waterlogging-tolerant squash plants. Our results could provide information for selecting the lines with the best tolerance to waterlogging-induced stress through breeding programs. Waterlogging-tolerant squash plants (i.e., EP cultivar) must be able to limit damages caused by stress to repairable levels, maintain physiological integrity, and mobilize mechanisms upon recovery to repair damages. More work needs to be conducted to confirm the effect of waterlogging on the production of EP plants under waterlogging conditions. In addition, relative waterlogging tolerance level in a genotype can change depending on the susceptible genotype. Therefore, further research is also needed to determine whether EP plants found to be tolerant in this study are significantly more tolerant than other pumpkin and squash lines or varieties.

The routes for light energy absorbed by Chl molecules in leaves are used to drive photosynthetic processes, dissipate heat, and re-emit light energy, and measuring the yield of ChlF gives specific information about photochemical efficiency and heat dissipation [24]. Under the stress of waterlogging, the ChlF, PRI, and photochemical reactions decline. The PRI is significantly correlated with the quantum yield of electron transfer in PSII and is indicative of xanthophyll cycle-mediated thermal energy dissipation. Indices using spectral bands were suggested for estimating the photosynthetic rate [25]. The *Fv/Fm* reduction indicates that an important portion of the PSII reaction center was damaged, and the Fv/Fm value in healthy leaves is close to 0.8, which is a typical value for uninhibited plants [26]. A lower value indicates that some proportion of the PSII reaction centers are damaged, which is often observed in plants under stressful conditions. When treatments were compared across stress times at 13 d, the EP plant (0.754 ± 0.042) exhibited significantly higher *Fv/Fm* values than SM (0.636 ± 0.077) and OK (0.347 ± 0.013) plants, indicating that EP is a waterlogging-tolerant cultivar. The Fv/Fm values in both SM and OK plants displayed significant decreases in waterlogging stress treatments at 13 d compared with controls, indicating that this parameter is suitable for evaluating the growth of these plants under waterlogging stress and suggesting a photo-inhibitory effect [27,28]. Furthermore, the YII values of OK plants under waterlogging treatment for 3 d (0.724 ± 0.020) were significantly lower compared with control under an identical time period (0.668 ± 0.030), whereas the values of SM plants under stress as late as the 13-d time point (0.481 ± 0.070) were found to be significantly lower than their control plants (0.622 ± 0.064). This finding, in turn, can yield useful information in determining which species can be targeted to be planted in zones with various periods of waterlogging. In the same way, an analysis of growth rate under waterlogging conditions can also be performed using the proposed non-destructive techniques. *Fv/Fm* and YII are widely used as indicators of the degree of plant stress and as criteria for genotype selection. Decreases in both parameters are associated with greater heat release in the antenna complex and the reduction in the amount of light energy used for photosynthesis increases photo-oxidative damage [29]. In this work, *Fv/Fm* and YII decreased under waterlogging conditions, indicating that PSII was damaged during the waterlogging stress. The stress level during waterlogging influences the photosynthetic potential of squash plants, and different responses of RWC, EL, and CC in leaves depend on the genotypes, which can be used to optimize the growth and development of plants in controlled waterlogging settings. There were significant correlations of PRI with *Fv/Fm* and YII in all plants, indicating that these reflectance indices can be useful for non-destructive estimations of leaf CC and ChlF in squashes. Automated, low-cost NDVI (>0.737 at 7-d waterlogging) and PRI (>0.049 at 13-d waterlogging) sensors offer new opportunities for monitoring photosynthetic phenology. This work demonstrated the feasibility of using spectral reflectance indices of waterlogging stress in squash plants to determine the levels of physiological parameters. The systems may be useful when screening for waterlogging-tolerant plants, for developing management practices for its cultivation in fields, and for enhancing cultivation during waterlogging.

Heavy rainfall in summer results in loss of fresh market production of squash plants. The knowledge of proper floodwater management can help formulate management options for enhancing and stabilizing plant survival and ensuring proper crop establishment in waterlogged soils. Our results suggest that the non-destructive parameters were stress-specific and not expressed solely in response to an increasing excess of photon energy. Singly or in combination, these indices provide a means to estimate photosynthetic phenology with the exact use depending upon the particular application and time frame. Thus, the use of non-destructive approaches should facilitate rapid and reliable field evaluation of a greater number of genotypes without compromising the selection accuracy of a traditional visual evaluation and destructive type of analysis. Combining SPAD (>50.27 at 3-d waterlogging), NVDI (>0.737 at 7-d waterlogging), YII (>0.670 at 13-d waterlogging), and *Fv/Fm* (>0.754 at 13-d waterlogging) values can be used to select against the most susceptible plants (i.e., OK cultivar), resulting in a more efficient use of land for evaluating the most tolerant plants (i.e., EP cultivar) or a new material of stands in the field. In addition, the average time required to measure SPAD, NVDI, YII, and *Fv/Fm* is very short. This means that hundreds of individual plants grown under the stress of waterlogging can be cost-effectively screened daily, providing ample opportunity to discover individuals that manifest better indicators and exhibit greater plant growth and photosynthetic apparatus in response to waterlogging. These findings are important for farming infrequently flooded areas, and are informative for further genetic and physiological studies of waterlogging tolerance in squashes. Adaption mechanisms of the squash plants are to be exploited in future squash breeding efforts.

## 4. Materials and Methods 

### 4.1. Plant Materials, Cultivation, and Waterlogging Treatments

Seeds of one Cma and two Cmo cultivars were purchased from Known-You Seed Co., Ltd. (Taipei, Taiwan). OK-101 (OK), a Cma species, is a new variety grown in Taiwan for consumption as a fresh vegetable that has black skin and an oblate shape with orange flesh. Early Price (EP), a Cmo species, is one of the most popular cultivars in Taiwan for consumption as a fresh vegetable that has an oblate shape with orange flesh and a brown rind. Strong Man (SM), a Cmo species, is a cold-tolerant variety being used as a grafting rootstock for watermelon and bitter melon in Taiwan. Culture practices, including watering and fertilization application, were described in our previous paper [30]. Briefly, the water imbibition seeds were germinated at 40 °C for three days. The germinated seeds were sown in a commercial potting mix of sand, peat moss, and perlite (1:1:1, *v/v/v*; Known-You Co., Taipei, Taiwan) and grown in a growth chamber under 350 μmol m^−2^ s^−1^ light with a 16-h photoperiod. The temperature of the tested plants was maintained at 25 °C (day) and 20 °C (night), and the relative humidity was maintained at 70%. Plants were watered with a half-strength Hoagland solution [31] with 500 mL^−1^ to each pot every other day and grown for 30 days (d) before imposition of waterlogging stress. The test pot plants were maintained by irrigation with 100 mL^−1^ tap water to each pot every day for 13 d (normal condition as control, C) or were subjected to waterlogging treatments for periods of 1, 3, 7, and 13 d. Water was drained out after 13 d of waterlogging stress and then the plants were allowed to recover for 3 d. The experiment followed a completely randomized design. There was a total of twelve treatments, i.e., three different genotypes with four different waterlogging time periods. Ten plants of each treatment per genotype were used for the analysis of SPAD, NDVI, and PRI values. Four plants of each treatment per genotype were used for relative water content (RWC), electrolyte leakage (EL), fresh weight, and dry weight measurements. All pots for each waterlogging time for the treatment group were randomly placed in 28 × 14 × 14 cm plastic buckets and subjected to waterlogging by filling the buckets with tap water to 2 cm above the soil surface. At different points in time following waterlogging, plants were taken out of the soil, and their roots were extensively washed in distilled water. Roots and leaves from each plant were clipped at the same time of the day, and immediately used for analyses. All one-month-old tested plants were used for phenotypes and various physiological parameter measurements in accordance with the duration of the stress period and subsequent recovery period. 

### 4.2. Plant Growth Measurements

Fresh weight (FW) of shoots and roots were measured as green shoot and roots, which were clipped at the soil surface to assess biomass accumulation. The dry weight (DW) of shoots and roots was measured after drying the shoots and roots in an oven at 70 °C for 48 h before weighing. The FW and DW of shoots and roots were measured with an electronic balance. The dry matter (DM, %) of shoots and roots was calculated as (DW/FW) × 100%. Data were collected at 13 d after treatment and 3 d post-waterlog recovery period after water drainage. Four plants per treatment were measured. After 13 d of waterlogging, most SM and OK plants were severely damaged, preventing the ability to collect leaf samples for the analysis of physiological parameters.

### 4.3. Relative Water Content (RWC)

Leaves were recut by a sharp razor blade and then immediately weighed (FW). In order to obtain the turgid mass, leaves were immersed in distilled water inside a closed Petri dish for 24 h. After gently wiping the water from the leaf surface with a tissue paper, the leaves were weighed (TW). Leaf samples were placed at 70 °C for 48 h in order to obtain the dry weight (DW). RWC (%) was calculated as [(FW − DW)/(TW − DW)] × 100% [32]. Data were collected at 13 d after treatment and 3 d post-waterlogging recovery period after water drainage. One leaf per plant and four plants per treatment were measured.

### 4.4. Determination of Electrolyte leakage (EL), Chlorophyll Content (CC), Chlorophyll Fluorescence (ChlF), and Spectral Reflectance

The cell membrane stability was estimated by measuring the electrolyte leakage of leaves according to the method of Huang and Guo [33]. Leaves were excised and immersed in 15 mL of distilled water in test tubes overnight at room temperature. The initial conductivity of the water was determined using a conductivity meter (model CDM 210, Radiometer, Cedex, France). Tubes were placed in boiling water for 15 min and then cooled to room temperature. The relative EL (%) was calculated as the ratio of conductivity before boiling to that after boiling. Data were collected at 13 d after treatment and 3 d post-waterlogging recovery period after water drainage. One leaf per plant and four plants per treatment were measured.

The CC was determined using a SPAD (soil and plant analysis development) analyzer (SPAD-502 Chlorophyll Meter, Konica Minolta, Tokyo, Japan). The SPAD value represents the relative CC of each leaf. Healthy, fully expanded third leaves were inserted into the measuring head and three readings were taken from each leaf and averaged. Data were collected from one leaf per plant and ten plants per treatment.

Potted plants were moved into the shade under a cottage before sunrise at 05:30–06:00, and then the ChlF parameters of the dark-adapted leaves were measured with a fluorometer (MINI-PAM, Heinz Walz, Effeltrich, Germany) at ambient temperature after adaptation to the dark for 30 min [34]. The middle portions of plants of both varieties were used for measurements. The values of minimal ChlF (Fo) and maximal ChlF (Fm) of dark-adapted samples were respectively determined using the modulated irradiation of a weak LED beam (measuring light) and a saturating pulse. We then calculated the maximum photochemical quantum yield (*Fv/Fm*), where Fv, the yield of variable fluorescence, was calculated as (Fm − Fo). When measuring *Fv/Fm*, samples were first acclimated to dark conditions to ensure that all reaction centers were in an open state and there was minimal non-photochemical dissipation of excitation energy. The yield of quantum efficiency (YII or the actual PSII efficiency, ΔF/Fm’) was calculated as (Fm′ − Ft)/Fm′. Fm′ and Ft are the maximal and steady-state levels of fluorescence during each level of illumination, respectively [35]. Measurements were recorded using the Win Control-3 software (Heinz Walz, Effeltrich, Germany). Data were collected at 1, 3, 7, and 13 d after treatment. One leaf per plant and ten plants per treatment were measured.

The spectral reflectance was measured from mature, healthy, fully expanded third leaves at wavelengths of 200–900 nm, using an integrating sphere fitted to a scanning spectrophotometer (Poly Pen RP 400, Photon Systems Instruments, Prague, Czech Republic). The following indices were calculated [36] from the reflectance spectrum: (1) NDVI, calculated as (R750 − R705)/(R750 + R705 − 2 × R445), was used to assess SPAD value and (2) PRI, calculated as (R531 − R570)/(R531 + R570), was used to assess both *Fv/Fm* and YII values.

### 4.5. Statistical Analysis

The measurements of phenotypes and physiological parameters were analyzed by a completely randomized design using one-way ANOVA that was performed to determine the significance of the differences among the responses of all genotypes to different waterlogging time points. For significant values, means were separated by the least significant difference (LSD) test at *p* ≤ 0.05 using SAS 9 (SAS Institute, Cary, NC, USA). The relationships among SPAD, ChlF component, and spectral reflectance were examined using regression analyses. In addition, model datasets were based on at least 10 pots from each waterlogging treatment and SPAD and ChlF components were calculated using spectral reflectance data from the model validation datasets. Several models were tested, including the linear and non-linear regression models that were selected for the best interpretation of the relationship between SPAD, ChlF component, and spectral reflectance. All models were evaluated for goodness of fit by the graphical analysis of higher R^2^ and lower mean square error.

## 5. Conclusions

In trying to understand the responses to waterlogging stress, physiological parameters induced by 1-, 3-, 7-, and 13-d periods of treatment were identified and characterized, and the effects of waterlogging on the growth and photosynthetic apparatus of three squash cultivars were examined. These genotypes exhibited unique abilities and specificities through the phenotype and physiological systems in response to the stress of waterlogging. Waterlogging for 13 d caused significant decreases of phenotypes in OK cultivar with lower shoot and root DW, FW, and DM, which led to physiological phenomena such as lower SPAD, *Fv/Fm*, YII, NDVI, and PRI values in leaves. Waterlogging caused higher physiological values with health maintenance in the EP cultivar and greater stress tolerance during waterlogging. The significant correlations among NDVI and PRI indices with SPAD, *Fv/Fm*, and YII can be used as selection indexes for examining the growth of squash plants at optimal waterlogging treatments. As a result, waterlogging-tolerant plants can be successfully selected based on these non-invasive estimations. The precise management of these parameters in response to waterlogging conditions can maximize the plant growth and Chl content of squash plants grown in controlled waterlogging environments.

## Figures and Tables

**Table 1:** Fresh weight, dry weight, and dry matter of shoots and roots, and root/shoot ratio of both fresh weight and dry weight in three squash cultivars (*Cucurbita moschata* cv. Early Price (EP), *Cucurbita moschata* cv. Strong Man (SM), and *Cucurbita maxima* cv. OK-101 (OK)) exposed to non-waterlogging (control, C) or waterlogging (W) for 13 days followed by drainage and recovery for 3 days.

					Recover	
Phenotype	Tissue	Cultivar	C	W	C	W
Fresh weight (g)	shoot	EP	11.01 ± 1.17 ^a^	9.07 ± 1.57 ^bc^	9.16 ± 1.04 ^bc^	8.98 ± 1.29 ^bc^
		SM	9.63 ± 1.89 ^bc^	9.01 ± 1.68 ^bc^	7.40 ± 0.40 ^cd^	8.40 ± 1.81 ^bcd^
		OK	10.87 ± 1.90 ^ab^	8.54 ± 1.02 ^bcd^	8.09 ± 1.63 ^bcd^	7.16 ± 1.16 ^d^
	root	EP	1.82 ± 0.90 ^bc^	2.45 ± 0.61 ^bc^	1.55 ± 0.57 ^cd^	3.73 ± 0.35 ^a^
		SM	1.34 ± 0.76 ^cd^	2.37 ± 0.74 ^bc^	1.31 ± 0.24 ^cd^	3.16 ± 0.74 ^ab^
		OK	1.41 ± 0.67 ^cd^	2.33 ± 0.16 ^bc^	1.12 ± 0.34 ^d^	1.62 ± 0.29 ^cd^
Dry weight (g)	shoot	EP	1.07 ± 0.28 ^cd^	1.53 ± 0.37 ^bc^	1.37 ± 0.42 ^bc^	1.79 ± 0.20 ^a^
		SM	0.77 ± 0.29 ^d^	0.77 ± 0.41 ^d^	1.37 ± 0.34 ^bc^	1.60 ± 0.51 ^ab^
		OK	0.84 ± 0.18 ^d^	1.21 ± 0.20 ^cd^	0.81 ± 0.27 ^d^	1.45 ± 0.42 ^bc^
	root	EP	0.16 ± 0.06 ^bcd^	0.17 ± 0.03 ^bc^	0.13 ± 0.07 ^bcd^	0.27 ± 0.05 ^a^
		SM	0.09 ± 0.06 ^de^	0.19 ± 0.07 ^b^	0.12 ± 0.03 ^bcd^	0.28 ± 0.11 ^a^
		OK	0.09 ± 0.05 ^de^	0.12 ± 0.03 ^bcd^	0.07 ± 0.03 ^e^	0.11 ± 0.04 ^cde^
Dry matter (%)	shoot	EP	9.56 ± 1.24 ^fg^	16.76 ± 2.99 ^cd^	18.45 ± 2.91 ^bc^	22.84 ± 2.86 ^a^
		SM	8.14 ± 0.81 ^g^	16.87 ± 3.14 ^cd^	14.86 ± 2.72 ^de^	20.15 ± 2.56 ^a^
		OK	7.73 ± 0.72 ^g^	13.45 ± 0.57 ^de^	10.06 ± 1.00 ^f^	14.54 ± 2.34 ^de^
	root	EP	7.16 ± 1.30 ^b^	6.97 ± 0.73 ^bc^	8.53 ± 1.62 ^ab^	11.58 ± 1.69 ^a^
		SM	6.20 ± 1.39 ^bc^	7.85 ± 1.18 ^ab^	9.10 ± 1.07 ^ab^	7.36 ± 1.86 ^b^
		OK	5.79 ± 0.75 ^c^	5.62 ± 1.12 ^c^	5.92 ± 1.54 ^c^	6.55 ± 1.10 ^bc^
Root/shoot FW (%)		EP	20.01 ± 3.51 ^c^	26.85 ± 2.71 ^b^	16.63 ± 4.35 ^cd^	42.16 ± 4.44 ^a^
		SM	14.61 ± 4.43 ^de^	27.72 ± 4.79 ^b^	17.80 ± 3.97 ^cd^	42.97 ± 4.32 ^a^
		OK	12.83 ± 2.13 ^e^	24.95 ± 3.74 ^bc^	14.27 ± 3.40 ^de^	20.29 ± 2.16 ^c^
Root/shoot DW (%)		EP	14.68 ± 2.62 ^b^	11.49 ± 2.79 ^bc^	9.41 ± 2.73 ^cd^	19.33 ± 3.38 ^a^
		SM	11.14 ± 3.07 ^bc^	13.95 ± 3.77 ^bc^	8.69 ± 2.18 ^cd^	15.77 ± 3.21 ^b^
		OK	9.68 ± 2.18 ^cd^	9.96 ± 1.61 ^bcd^	8.37 ± 2.49 ^cd^	8.02 ± 1.47 ^d^

Among the three cultivars of the same tissue under C and W treatments in each phenotype measurement, means with same letters are not significantly different by the least significant difference (LSD) at *p* ≤ 0.05 with a completely randomized design. Each value is the mean of four plants of each cultivar for C or W treatment.

**Table 2:** Effects of waterlogging on soil and plant analysis development (SPAD) chlorophyll meter value in EP, SM, and OK squash plants. Plants were grown under optimally irrigated (control, C) or waterlogging (W) conditions for different time periods.

			Duration (day)				
Index	Cultivar	Treatment	0	1	3	7	13
SPAD	EP	C	46.24 ± 3.27 ^bc^	49.58 ± 2.13 ^bc^	53.42 ± 4.07 ^a^	52.11 ± 2.02 ^a^	51.78 ± 4.27 ^ab^
		W	46.54 ± 3.36 ^bc^	49.31 ± 2.72 ^bc^	50.27 ± 2.94 ^ab^	47.05 ± 2.03 ^bc^	41.04 ± 3.22 ^cd^
	SM	C	42.14 ± 3.31 ^bcd^	41.74 ± 3.65 ^bcd^	43.86 ± 3.32 ^bc^	42.96 ± 2.05 ^bc^	40.77 ± 3.67 ^cd^
		W	42.02 ± 3.57 ^bcd^	39.23 ± 3.70 ^cde^	38.51 ± 2.62 ^cde^	30.59 ± 3.05 ^de^	10.35 ± 2.02 ^g^
	OK	C	29.21 ± 3.51 ^de^	28.11 ± 3.16 ^de^	30.31 ± 3.87 ^de^	31.00 ± 4.09 ^de^	30.77 ± 2.09 ^de^
		W	29.41 ± 3.90 ^de^	28.81 ± 3.64 ^de^	26.20 ± 2.93 ^ef^	23.75 ± 2.55 ^f^	6.45 ± 1.16 ^h^

Different letters indicate significant difference among various waterlogging treatments of all cultivars (*p* < 0.05). Each value is the mean of ten plants of each cultivar for C or W treatment. Data were collected at 0 (non-waterlogging), 1, 3, 7, and 13 days of waterlogging treatment.

**Table 3:** Effects of waterlogging on variable/maximal fluorescence (*Fv/Fm*) and quantum photosynthetic yield (YII) values in EP, SM, and OK squash plants. Plants were grown under optimally irrigated (control, C) or waterlogging (W) conditions for different time periods.

			Duration (day)				
Index	Cultivar	Treatment	0	1	3	7	13
Fv/Fm	EP	C	0.798 ± 0.006 ^ab^	0.798 ± 0.008 ^ab^	0.798 ± 0.009 ^ab^	0.797 ± 0.006 ^ab^	0.792 ± 0.012 ^ab^
		W	0.795 ± 0.007 ^ab^	0.800 ± 0.014 ^a^	0.790 ± 0.013 ^ab^	0.768 ± 0.033 ^ab^	0.754 ± 0.042 ^b^
	SM	C	0.794 ± 0.014 ^ab^	0.795 ± 0.006 ^ab^	0.800 ± 0.007 ^a^	0.803 ± 0.010 ^a^	0.790 ± 0.018 ^ab^
		W	0.794 ± 0.006 ^ab^	0.795 ± 0.009 ^ab^	0.771 ± 0.020 ^ab^	0.776 ± 0.023 ^ab^	0.636 ± 0.077 ^c^
	OK	C	0.807 ± 0.007 ^a^	0.810 ± 0.007 ^a^	0.810 ± 0.006 ^a^	0.802 ± 0.007 ^a^	0.788 ± 0.025 ^ab^
		W	0.804 ± 0.007 ^a^	0.803 ± 0.007 ^a^	0.802 ± 0.009 ^a^	0.790 ± 0.014 ^ab^	0.347 ± 0.013 ^d^
Y II	EP	C	0.683 ± 0.055 ^bc^	0.673 ± 0.022 ^bc^	0.685 ± 0.042 ^bc^	0.680 ± 0.043 ^bc^	0.645 ± 0.026 ^c^
		W	0.686 ± 0.044 ^bc^	0.642 ± 0.012 ^c^	0.671 ± 0.038 ^bc^	0.644 ± 0.058 ^c^	0.670 ± 0.055 ^bc^
	SM	C	0.688 ± 0.034 ^bc^	0.662 ± 0.025 ^bc^	0.668 ± 0.032 ^bc^	0.670 ± 0.042 ^bc^	0.622 ± 0.064 ^c^
		W	0.677 ± 0.024 ^bc^	0.647 ± 0.014 ^c^	0.656 ± 0.061 ^c^	0.649 ± 0.048 ^c^	0.481 ± 0.070 ^d^
	OK	C	0.706 ± 0.025 ^ab^	0.684 ± 0.017 ^bc^	0.724 ± 0.020 ^a^	0.704 ± 0.028 ^ab^	0.692 ± 0.019 ^bc^
		W	0.708 ± 0.028 ^ab^	0.681 ± 0.011 ^bc^	0.668 ± 0.030 ^bc^	0.644 ± 0.044 ^c^	0.237 ± 0.013 ^e^

Different letters indicate significant difference among various waterlogging treatments of all cultivars in each index (*p* < 0.05). Each value is the mean of ten plants of each cultivar for C or W treatment. Data were collected at 0, 1, 3, 7, and 13 days of waterlogging treatment.

**Table 4:** Effects of waterlogging on normalized difference vegetation index (NDVI) and photochemical reflectance index (PRI) values in EP, SM, and OK squash plants. Plants were grown under optimal irrigated (control, C) or waterlogging (W) conditions for different time periods.

			Duration (day)				
Index	Cultivar	Treatment	0	1	3	7	13
NDVI	EP	C	0.739 ± 0.009 ^ab^	0.736 ± 0.008 ^ab^	0.738 ± 0.007 ^ab^	0.731 ± 0.010 ^ab^	0.731 ± 0.015 ^ab^
		W	0.744 ± 0.007 ^a^	0.737 ± 0.011 ^ab^	0.746 ± 0.014 ^a^	0.737 ± 0.011 ^ab^	0.711 ± 0.016 ^bc^
	SM	C	0.734 ± 0.017 ^ab^	0.736 ± 0.008 ^ab^	0.739 ± 0.008 ^ab^	0.733 ± 0.017 ^ab^	0.727 ± 0.016 ^ab^
		W	0.735 ± 0.014 ^ab^	0.739 ± 0.011 ^ab^	0.732 ± 0.011 ^ab^	0.686 ± 0.021 ^de^	0.376 ± 0.017 ^h^
	OK	C	0.703 ± 0.019 ^cd^	0.697 ± 0.025 ^de^	0.706 ± 0.014 ^cd^	0.709 ± 0.018 ^cd^	0.699 ± 0.020 ^de^
		W	0.704 ± 0.017 ^cd^	0.689 ± 0.020 ^de^	0.681 ± 0.039 ^de^	0.648 ± 0.021 ^f^	0.459 ± 0.080 ^g^
PRI	EP	C	0.065 ± 0.007 ^bc^	0.057 ± 0.008 ^c^	0.054 ± 0.010 ^c^	0.052 ± 0.007 ^c^	0.051 ± 0.010 ^cd^
		W	0.064 ± 0.005 ^bc^	0.064 ± 0.005 ^bc^	0.057 ± 0.006 ^c^	0.065 ± 0.004 ^bc^	0.049 ± 0.004 ^d^
	SM	C	0.065 ± 0.009 ^bc^	0.064 ± 0.004 ^bc^	0.065 ± 0.008 ^bc^	0.063 ± 0.007 ^bc^	0.061 ± 0.008 ^bc^
		W	0.065 ± 0.009 ^bc^	0.068 ± 0.006 ^bc^	0.067 ± 0.005 ^bc^	0.060 ± 0.006 ^bc^	0.024 ± 0.013 ^e^
	OK	C	0.067 ± 0.004 ^bc^	0.064 ± 0.004 ^bc^	0.067 ± 0.006 ^bc^	0.072 ± 0.004 ^a^	0.063 ± 0.009 ^bc^
		W	0.068 ± 0.002 ^bc^	0.067 ± 0.003 ^bc^	0.062 ± 0.004 ^bc^	0.064 ± 0.004 ^bc^	0.018 ± 0.013 ^e^

Different letters indicate significant difference among various waterlogging treatments of all cultivars in each index (*p* < 0.05). Each value is the mean of ten plants of each cultivar for C or W treatment. Data were collected at 0, 1, 3, 7, and 13 days of waterlogging treatment.
